# Early Postoperative Dupilumab After Revision Endoscopic Sinus Surgery for CRSwNP: A Real-World Single-Centre Study

**DOI:** 10.3390/jcm15083015

**Published:** 2026-04-15

**Authors:** Juan David Gutiérrez-Posso, Aitor Zabala-López de Maturana

**Affiliations:** 1 Otorhinolaryngology Department, Basurto University Hospital, OSI-Bilbao Basurto, 48013 Bilbao, Spain; javieraitor.zabalalopezmaturana@osakidetza.eus; 2 BioBizkaia Health Research Institute, 48903 Barakaldo, Spain

**Keywords:** chronic rhinosinusitis with nasal polyps, dupilumab, biologic therapy, endoscopic sinus surgery, postoperative treatment, real-world study, nasal polyps, type 2 inflammation

## Abstract

**Background/Objectives:** Chronic rhinosinusitis with nasal polyps (CRSwNP) is a type 2 inflammatory disease frequently associated with recurrence after endoscopic sinus surgery (ESS). Although biologic therapies such as dupilumab have demonstrated efficacy in severe CRSwNP, the optimal timing of treatment initiation in relation to surgery remains unclear. This study aimed to evaluate the clinical outcomes of early postoperative initiation of dupilumab after revision ESS using a multidimensional assessment of disease control. **Methods:** This retrospective observational study included adult patients with severe CRSwNP treated with dupilumab at a tertiary referral centre. All patients had undergone at least two previous ESS procedures and initiated dupilumab within 30 days following revision surgery. Clinical outcomes were assessed at baseline and after 12 months, including Nasal Polyp Score (NPS), Sinonasal Outcome Test-22 (SNOT-22), nasal congestion and olfactory visual analogue scale (VAS) scores, and asthma control in patients with comorbid asthma. Treatment response was evaluated using a multidomain assessment. **Results:** Ten patients were included. After 12 months, significant improvements were observed in NPS (from 4.7 ± 2.3 to 0.4 ± 1.0; *p* = 0.0019) and SNOT-22 (from 61.9 ± 17.3 to 26.5 ± 14.7; *p* = 0.0019). Nasal congestion and olfactory VAS scores also improved significantly. Most patients (70%) achieved an excellent multidimensional response, while 30% showed a moderate response. No patients required systemic corticosteroids or revision surgery during follow-up. **Conclusions:** Early postoperative initiation of dupilumab after revision ESS was associated with improvements in endoscopic findings, symptom severity, and quality of life. These findings suggest that the early postoperative period may represent a therapeutic window in selected patients with severe recurrent CRSwNP. However, results should be interpreted with caution and considered hypothesis-generating.

## 1. Introduction

Chronic rhinosinusitis with nasal polyps (CRSwNP) is a chronic inflammatory disease of the upper airways predominantly driven by type 2 immune mechanisms and typically managed with intranasal corticosteroids and saline irrigations [1]. The most burdensome patient-reported symptoms include nasal obstruction, rhinorrhea, and olfactory dysfunction, which significantly impair quality of life and daily functioning [2]. Despite appropriate medical therapy, a proportion of patients remains uncontrolled and requires repeated courses of systemic corticosteroids as well as endoscopic sinus surgery (ESS).

Although ESS effectively reduces local inflammatory burden by removing nasal polyps and improving sinus ventilation, it does not directly modify the underlying type 2 inflammatory endotype responsible for disease persistence and recurrence [3]. Consequently, recurrence after surgery remains frequent, particularly in patients with persistent type 2 inflammation and those who have undergone multiple surgical procedures.

In recent years, monoclonal antibodies targeting key mediators of type 2 inflammation have expanded treatment options for patients with uncontrolled CRSwNP. These biologic therapies act by blocking specific inflammatory pathways, including IL-4/IL-13 signaling (dupilumab), immunoglobulin E (omalizumab), and interleukin-5 (mepolizumab) [4,5,6]. Randomized clinical trials have demonstrated that these therapies significantly reduce nasal polyp size, improve symptom burden, and decrease the need for systemic corticosteroids and revision surgery in patients with severe CRSwNP.

Beyond randomized trials, real-world studies have further supported the effectiveness of biologic therapies in routine clinical practice. Several observational studies evaluating anti-IL-5 therapies have reported improvements in endoscopic findings, patient-reported outcomes, and disease control in patients with refractory CRSwNP [7,8,9,10]. Similarly, multicentre observational studies have confirmed the effectiveness of dupilumab in patients with severe uncontrolled disease, demonstrating substantial improvements in symptom severity and quality of life in real-life clinical settings [11].

Despite the growing evidence supporting biologic therapies in CRSwNP, current recommendations mainly focus on defining treatment indications and patient selection criteria. However, the optimal timing for initiating biologic therapy in relation to sinus surgery remains unclear. In particular, evidence regarding early postoperative initiation of biologic therapy in patients with post-surgical CRSwNP is still limited in real-world clinical practice.

Therefore, the aim of the present study was to evaluate the clinical outcomes of early postoperative dupilumab initiation in patients with post-surgical CRSwNP using a multidimensional assessment integrating endoscopic findings, patient-reported outcomes, and biological parameters in a real-world single-centre cohort.

## 2. Materials and Methods

### 2.1. Study Design and Patient Selection

This retrospective observational study was conducted at a tertiary referral hospital between 2024 and 2025 and included adult patients with chronic rhinosinusitis with nasal polyps (CRSwNP) who initiated dupilumab therapy in the early postoperative period following revision endoscopic sinus surgery (ESS) in routine clinical practice.

In accordance with POLINA recommendations [12], all included patients had undergone at least two previous endoscopic sinus surgeries before initiation of dupilumab therapy. In our institution, biologic treatment is considered in patients with recurrent disease after repeated surgery and evidence of persistent type 2 inflammation.

Inclusion criteria were: age ≥ 18 years; diagnosis of severe CRSwNP, supported by a Nasal Polyp Score (NPS) ≥ 5 and/or Sinonasal Outcome Test (SNOT-22) ≥ 50; evidence of type 2 inflammation, defined by blood eosinophil counts > 300 cells/μL, tissue eosinophils ≥ 10 per high-power field (HPF), and/or total IgE ≥ 100; persistent symptoms despite treatment with intranasal corticosteroids; and failure of, contraindication to, or intolerance of previous medical treatments, including at least two courses of systemic corticosteroids in the previous year and/or a history of ≥2 ESS procedures, in line with current recommendations (POLINA and EPOS/EUFOREA). Exclusion criteria were age <18 years, pregnancy or breastfeeding.

As part of routine clinical practice in our institution, dupilumab was administered at a dose of 300 mg subcutaneously every 14 days. Treatment was initiated within the first 30 days following revision ESS. The decision to initiate biologic therapy was made by a multidisciplinary team including otolaryngologists, allergists, pulmonologists, and hospital pharmacists.

Patients were scheduled for follow-up visits at 6 and 12 months after initiation of dupilumab therapy. Treatment adherence was assessed at each visit; patients recorded the date of each injection using a mobile device and reported this information during follow-up. In addition, patients were systematically asked about adverse events at each visit, and any reported events were documented.

Patients were consecutively included, and clinical data were retrospectively extracted from electronic medical records. Baseline values for clinical, laboratory, and patient-reported outcomes corresponded to those recorded within the 30 days prior to initiation of dupilumab treatment. All patients had a minimum follow-up of 12 months, and clinical outcomes were evaluated at this time point. Patients receiving other biologic therapies or lacking sufficient follow-up data were excluded.

Standard postoperative care included saline irrigations and intranasal corticosteroids according to institutional protocols. Dupilumab (Sanofi, Paris, France/Regeneron Pharmaceuticals, New York, NY, USA) was initiated once adequate mucosal healing was achieved and the postoperative cavity was considered clinically stable, typically during the third to fourth postoperative week.

The study was conducted in accordance with the principles of the Declaration of Helsinki and was approved by the institutional ethics committee of the participating centre (internal approval code: 109.25). Given the retrospective design and the use of anonymized data, the requirement for written informed consent was waived.

### 2.2. Clinical Endpoints

The effectiveness of dupilumab therapy was evaluated using a multidimensional assessment of treatment response, integrating objective endoscopic findings, patient-reported outcomes, comorbidity control, and the need for rescue treatment.

The primary endpoint was the change in Nasal Polyp Score (NPS) from baseline to 12 months after initiation of dupilumab therapy. Endoscopic evaluation was performed using the standard NPS grading system, in which each nasal cavity is scored from 0 (no polyps) to 4 (complete obstruction), resulting in a total score ranging from 0 to 8.

To capture the multidimensional impact of treatment, response was assessed across five clinical domains:

1. Endoscopic improvement, defined as a reduction in NPS of ≥1 point compared with baseline.
2. Quality of life improvement, defined as a reduction of ≥8.9 points in the SNOT-22 score, corresponding to the minimal clinically important difference.
3. Olfactory symptom improvement, defined as an increase of ≥3 points in the olfactory visual analogue scale (VAS).
4. Asthma control, defined as an increase of ≥3 points in the Asthma Control Test (ACT) score in patients with concomitant asthma.
5. Absence of rescue treatment, defined as no requirement for systemic corticosteroids or revision endoscopic sinus surgery during the follow-up period.

Because not all patients had a diagnosis of asthma, the ACT domain was only evaluated in patients with concomitant asthma. In patients without asthma, this domain was considered not applicable and was excluded from the multidimensional evaluation.

For each patient, the number of domains meeting the predefined response criteria was calculated. Based on the number of fulfilled domains, treatment response was categorized as follows:

- Excellent response: fulfillment of four or more domains;
- Moderate response: fulfillment of three domains;
- No response: fulfillment of two or fewer domains.

This multidomain clinical assessment was used to provide a comprehensive evaluation of treatment effectiveness across different aspects of disease control.

Six-month follow-up data were not included in the analysis because complete data were not available for all patients, particularly for patient-reported outcomes and laboratory parameters.

### 2.3. Statistical Analysis

Continuous variables are presented as mean ± standard deviation (SD). When appropriate, median and interquartile range (IQR) were also reported.

Changes between baseline and 12-month follow-up were analysed using paired statistical tests. Given the small sample size, the Wilcoxon signed-rank test was used to evaluate differences in continuous variables.

For the multidomain response assessment, the proportion of patients fulfilling each predefined response domain was calculated, and overall treatment response was determined according to the number of domains achieved by each patient. All statistical analyses were performed using STATA statistical software version 18 (StataCorp LLC, College Station, TX, USA). A two-sided *p*-value < 0.05 was considered statistically significant.

## 3. Results

### 3.1. Patient Characteristics

A total of 10 patients with chronic rhinosinusitis with nasal polyps (CRSwNP) treated with dupilumab were included in the study. All patients had previously undergone at least two endoscopic sinus surgeries (ESS) prior to initiation of biologic therapy in accordance with national eligibility criteria.

Dupilumab therapy was initiated within 30 days following revision ESS, typically between the third and fourth postoperative week (approximately days 21–28 after surgery). This timing reflected routine clinical practice in our centre, once the postoperative cavity had stabilized. All patients completed 12 months of follow-up.

Baseline demographic and clinical characteristics of the cohort are summarized in Table 1. The mean age of the study population was 57.6 ± 10.3 years, and the cohort included 5 men and 5 women.

Evidence of type 2 inflammatory disease was present in several patients. Asthma was diagnosed in 7 patients (70%), while 3 patients (30%) did not have asthma. In addition, 2 patients (20%) were diagnosed with NSAID-exacerbated respiratory disease (N-ERD). As expected, all patients with N-ERD also had concomitant asthma.

At baseline, patients presented a substantial disease burden, reflected by elevated nasal polyp scores, impaired quality of life, and relevant symptom severity, including nasal obstruction and olfactory dysfunction.

### 3.2. Changes in Clinical Outcomes After Dupilumab Therapy

Clinical outcomes at baseline and after 12 months of dupilumab therapy are summarized in Table 2.

**Figure 1:**
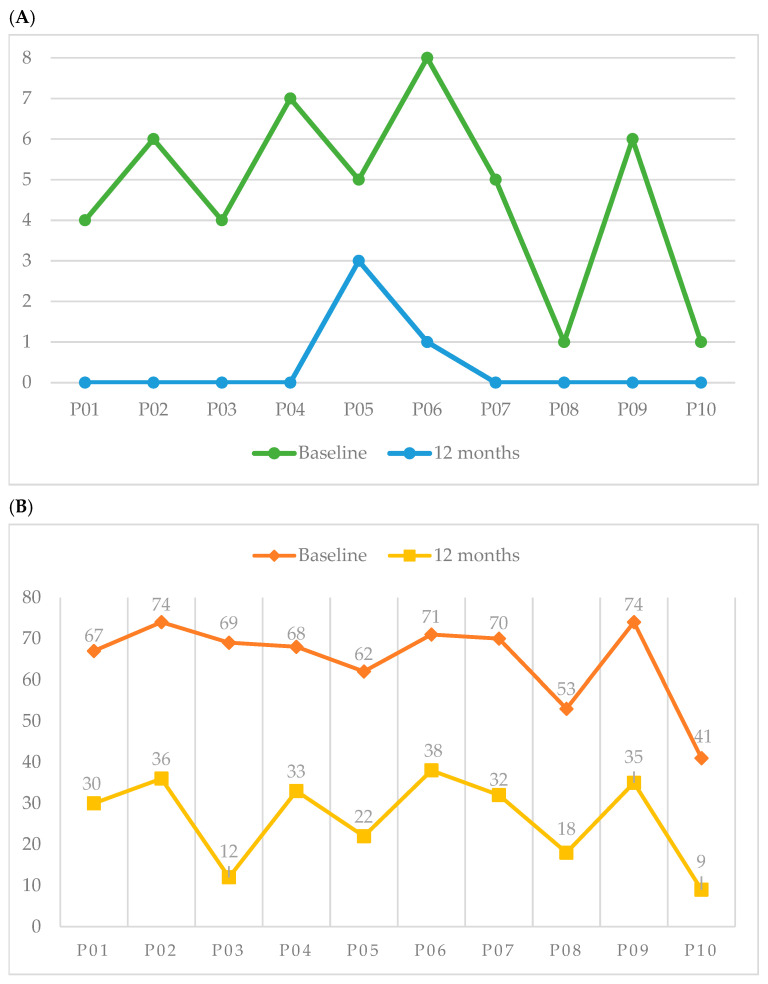
Individual changes in clinical outcomes after 12 months of dupilumab therapy. (**A**) Changes in Nasal Polyp Score (NPS) from baseline to 12 months. (**B**) Changes in SNOT-22 scores during the same period. Each line represents an individual patient, illustrating the consistent reduction in nasal polyp burden and the improvement in sinonasal-related quality of life after treatment.

A significant improvement in endoscopic disease severity was observed during follow-up. The mean Nasal Polyp Score (NPS) decreased from 4.7 ± 2.3 at baseline to 0.4 ± 1.0 at 12 months (*p* = 0.0019), as illustrated in Figure 1A, indicating a marked reduction in nasal polyp burden.

Similarly, quality of life improved substantially after treatment. The SNOT-22 score decreased from 61.9 ± 17.3 at baseline to 26.5 ± 14.7 after 12 months (*p* = 0.0019) (Figure 1B). The magnitude of this reduction exceeded the minimal clinically important difference (MCID) of 8.9 points for this instrument.

**Figure 2:**
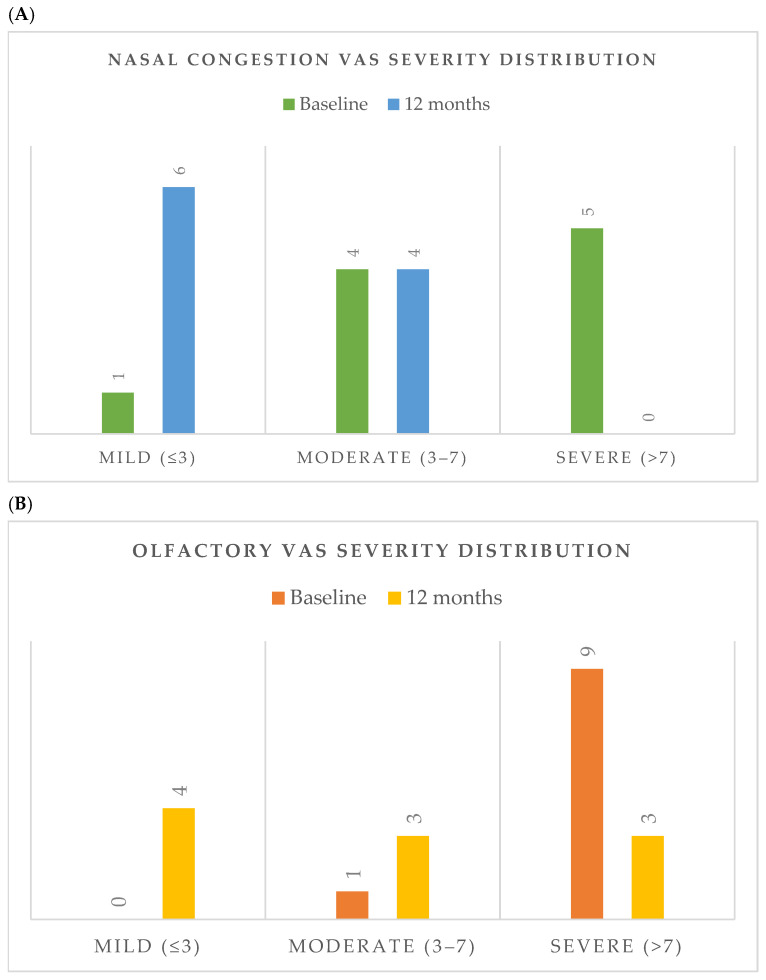
Distribution of symptom severity at baseline and after 12 months of dupilumab therapy. (**A**) Nasal congestion VAS severity distribution. (**B**) Olfactory VAS severity distribution. Severity categories were defined as mild (VAS ≤ 3), moderate (3 < VAS ≤ 7), and severe (VAS > 7). Abbreviations: VAS, visual analogue scale.

Improvements were also observed in symptom severity, as reflected by reductions in visual analogue scale (VAS) scores. Mean nasal congestion VAS decreased from 7.3 ± 2.2 to 2.5 ± 2.0 (*p* = 0.0078), while olfactory dysfunction VAS improved from 9.4 ± 1.1 to 4.6 ± 4.1 (*p* = 0.0156). The distribution of nasal congestion and olfactory dysfunction severity categories at baseline and after 12 months of treatment is shown in Figure 2A,B, showing a shift toward lower severity categories after treatment.

Peripheral blood eosinophil counts showed a numerical decrease from 404 ± 215 cells/µL to 342 ± 197 cells/µL, although this change did not reach statistical significance (*p* = 0.138).

Among patients with concomitant asthma (n = 7), Asthma Control Test (ACT) scores increased from 20.3 ± 5.9 at baseline to 23.9 ± 2.3 at 12 months, although the difference did not reach statistical significance (*p* = 0.375).

### 3.3. Multidimensional Response Analysis

To provide a comprehensive evaluation of treatment effectiveness, a multidimensional response analysis was performed integrating improvements across several domains of disease control. The evaluated domains included endoscopic improvement (NPS reduction ≥ 1), quality-of-life improvement (SNOT-22 reduction ≥ 8.9), improvement in olfactory symptoms (VAS increase ≥ 3), improvement in asthma control (ACT increase ≥ 3 in patients with asthma), and absence of rescue treatment during follow-up.

The proportion of patients fulfilling each response domain was calculated. A reduction of at least one point in Nasal Polyp Score was observed in all patients (10/10, 100%), corresponding to clinical improvement in endoscopic disease severity. Similarly, a clinically relevant improvement in SNOT-22 score (≥8.9 points) was achieved by all patients (10/10, 100%), indicating substantial improvement in sinonasal-related quality of life.

Improvement in olfactory symptoms was observed in 6 patients (60%). Among patients with concomitant asthma (n = 7), an increase of ≥3 points in ACT score was observed in 3 patients (42.9%), indicating improved asthma control in a subset of patients.

Importantly, all patients (10/10, 100%) remained free of rescue treatment during follow-up, with no requirement for systemic corticosteroids or revision endoscopic sinus surgery.

**Figure 3:**
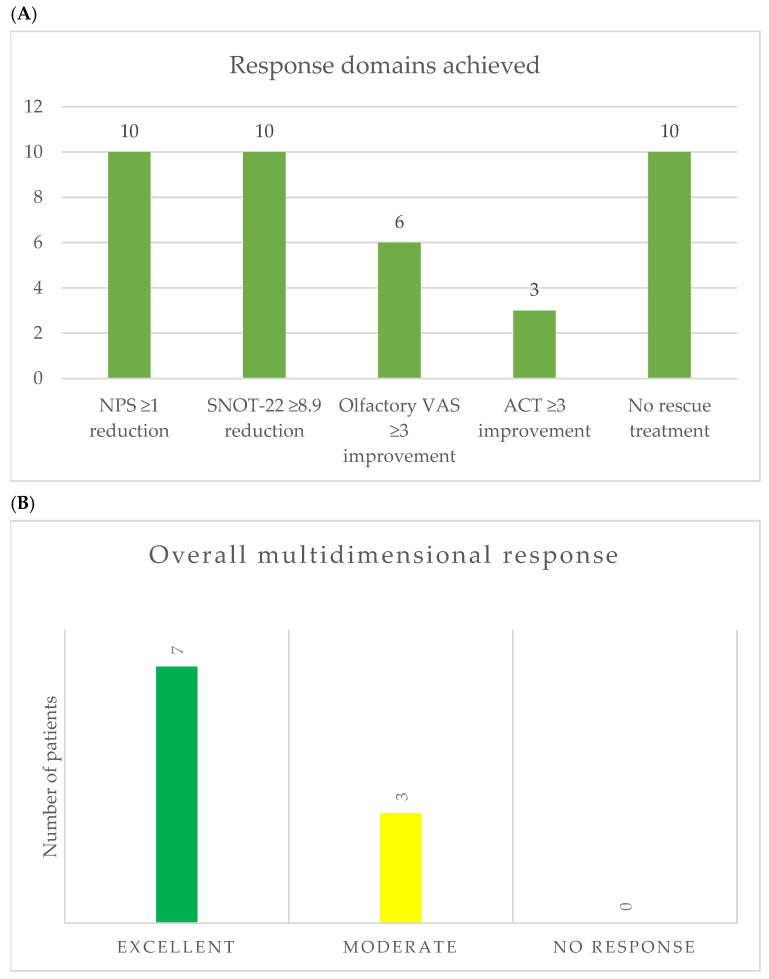
Multidimensional response to dupilumab after 12 months of therapy. (**A**) Number of patients achieving improvement across individual response domains, including reduction in nasal polyp score, improvement in SNOT-22, olfactory symptoms, asthma control, and absence of rescue treatment. (**B**) Overall multidimensional response according to the number of domains achieved.

Based on the number of response domains fulfilled, patients were categorized into predefined response groups. Overall, 7 patients (70%) achieved an excellent multidimensional response, fulfilling four or more domains, while 3 patients (30%) showed a moderate response, fulfilling three domains. No patients met the predefined criteria for non-response. The individual distribution of multidimensional responses is presented in Table 3, while the distribution of treatment response across the evaluated domains and the overall response categories are illustrated in Figure 3A,B, showing that most patients achieved improvement across multiple domains of disease control.

## 4. Discussion

This study evaluated the clinical outcomes associated with early postoperative initiation of dupilumab following revision endoscopic sinus surgery (ESS) in patients with severe chronic rhinosinusitis with nasal polyps (CRSwNP). After 12 months of treatment, marked improvements were observed in endoscopic findings, patient-reported outcomes, and symptom severity, and most patients achieved improvement across multiple domains of disease control.

The magnitude of improvement observed in our cohort is consistent with previous evidence on biologic therapies in CRSwNP. Both randomized and real-world studies have shown that dupilumab reduces nasal polyp burden and improves quality of life, symptom severity, and olfactory outcomes in patients with severe uncontrolled disease [13,14,15,16]. In our series, the reductions in NPS and SNOT-22 after 12 months were broadly in line with those reported in those studies, despite the fact that our patients represented a highly selected population with severe recurrent disease and multiple prior surgical procedures.

A key aspect of our study is the timing of biologic initiation. Dupilumab was started within the first month after revision ESS, typically during the third to fourth postoperative week. This strategy is biologically plausible because, although surgery reduces inflammatory load and restores sinus ventilation, it does not modify the underlying type 2 inflammatory endotype that drives recurrence [17].

The optimal timing of biologic therapy in relation to surgery remains insufficiently defined. However, available evidence suggests that combining both strategies within a short interval may influence outcomes. In a comparative study, patients undergoing ESS in close temporal proximity to biologic therapy showed a greater reduction in polyp burden than patients treated with biologic therapy alone, supporting a possible additive effect of both interventions [18].

These considerations are particularly relevant because CRSwNP is a multidimensional disease in which treatment response cannot be assessed only by endoscopic findings. Symptom burden, quality of life, smell, comorbidity control, and the need for rescue treatment are also essential components of disease control [19]. In addition, the high prevalence of asthma in our cohort supports the concept of united airway disease and reinforces the rationale for therapies targeting shared type 2 inflammatory pathways across the upper and lower airways [20].

Recent comparative studies also help to contextualize our findings. Real-world data comparing dupilumab with ESS have shown that both strategies are effective over 12 months, although with different response profiles: surgery may provide a greater early reduction in polyp burden, whereas dupilumab may offer greater improvement in quality of life, olfactory outcomes, local inflammatory control, and need for oral corticosteroids at later time points [21]. Similarly, a systematic review and meta-analysis comparing dupilumab with sinus surgery found that surgery was more effective during the first postoperative months for some outcomes, whereas at around 1 year both approaches became more similar, with greater olfactory improvement in the dupilumab group [22].

Further evidence from postoperative studies supports the clinical relevance of the perioperative window. A meta-analysis of real-world evidence confirmed that biologic therapies, particularly dupilumab, provide strong and sustained clinical benefit across multiple time points in routine practice [23]. In a retrospective cohort study, postoperative dupilumab used as an adjuvant to ESS was associated with greater reductions in endoscopic scores and NPS than surgery alone [24]. Likewise, an exploratory pilot study reported that adjuvant postoperative dupilumab improved immediate recovery, with better early endoscopic, SNOT-22, and olfactory outcomes than surgery alone, although this advantage was less evident by 6 months [25]. Finally, comparative real-life data on biologic response time suggest that dupilumab may achieve a faster endoscopic response than other available biologics, with significant NPS improvement already evident from the first weeks of treatment [26]. Taken together, these studies support the hypothesis that earlier postoperative modulation of type 2 inflammation may positively influence the short- and mid-term trajectory of disease control.

In this context, our findings add to the growing evidence suggesting that the early postoperative period may represent a therapeutic window in selected patients with severe recurrent CRSwNP. The marked reduction in polyp burden, the improvement across several clinical domains, and the absence of rescue treatment during follow-up suggest the feasibility of this strategy in real-world practice.

The interpretation of our findings should nevertheless remain cautious. Because dupilumab was introduced during the postoperative period and no comparator group was available, the relative contribution of surgery, postoperative care, and biologic therapy cannot be separated. Therefore, our results should be interpreted as descriptive of a real-world management strategy and as hypothesis-generating rather than as evidence of a proven causal treatment effect.

Several limitations should be acknowledged. The small sample size and single-centre design limit generalizability. In addition, the cohort represents a highly selected population with severe recurrent disease requiring multiple previous surgical procedures and meeting criteria for biologic therapy. The absence of a control group further limits interpretation and precludes direct comparison with alternative strategies, including surgery alone or delayed biologic initiation.

Despite these limitations, this study provides real-world evidence supporting the feasibility and potential value of early postoperative initiation of dupilumab in selected patients with severe recurrent CRSwNP. Larger prospective controlled studies are needed to better define the optimal timing of biologic therapy in relation to sinus surgery and to determine whether early combined approaches translate into improved long-term outcomes.

## 5. Conclusions

Early postoperative initiation of dupilumab after revision endoscopic sinus surgery was associated with improvements in endoscopic findings, symptom severity, and quality of life in this cohort of patients with severe recurrent CRSwNP.

These findings support the concept that the early postoperative period may represent a relevant therapeutic window for modulating type 2 inflammation and improving disease control in selected patients. However, given the small sample size, retrospective design, and absence of a control group, these findings should be interpreted with caution and considered hypothesis-generating.

Future prospective controlled studies are needed to compare early versus delayed biologic initiation strategies and to better define the optimal timing of biologic therapy in relation to sinus surgery.

## Figures and Tables

**Table 1:** Baseline characteristics of the study population.

Variable	n = 10
**Age (years)**	
Mean ± SD	57.6 ± 10.3
Median [IQR]	59.5 [50.0–65.8]
**Sex, n (%)**	
Male	5 (50%)
Female	5 (50%)
**Asthma, n (%)**	
No	3 (30%)
Yes	7 (70%)
**N-ERD, n (%)**	
No	8 (80%)
Yes	2 (20%)
**Number of previous ESS**	
≥2 surgeries	10 (100%)
**Peripheral blood eosinophils (cells/µL)**	
Mean ± SD	404 ± 215
Median [IQR]	420 [238–578]
**Nasal Polyp Score (NPS)**	
Mean ± SD	4.7 ± 2.3
Median [IQR]	5.0 [4.0–6.0]
**SNOT-22**	
Mean ± SD	61.9 ± 17.3
Median [IQR]	67.5 [56.8–70.8]
**Nasal congestion VAS**	
Mean ± SD	7.3 ± 2.2
Median [IQR]	7.5 [5.0–9.0]
**Olfactory VAS**	
Mean ± SD	9.4 ± 1.1
Median [IQR]	10.0 [9.3–10.0]

Abbreviations: ESS: endoscopic sinus surgery; N-ERD: NSAID-exacerbated respiratory disease; NPS: Nasal Polyp Score; SNOT-22: Sinonasal Outcome Test-22; VAS: visual analogue scale; IQR: interquartile range; SD: standard deviation.

**Table 2:** Clinical outcomes at baseline and after 12 months of dupilumab therapy.

Outcome	Baseline	12 Months	*p*-Value
**Total NPS**			
Mean ± SD	4.7 ± 2.3	0.4 ± 1.0	0.0019
Median [IQR]	5.0 [4.0–6.0]	0.0 [0.0–0.0]	
**SNOT-22**			
Mean ± SD	61.9 ± 17.3	26.5 ± 14.7	0.0019
Median [IQR]	67.5 [56.8–70.8]	30.5 [12.8–37.5]	
**Nasal congestion VAS**			
Mean ± SD	7.3 ± 2.2	2.5 ± 2.0	0.0078
Median [IQR]	7.5 [5.0–9.0]	2.0 [1.3–4.5]	
**Olfactory VAS**			
Mean ± SD	9.4 ± 1.1	4.6 ± 4.1	0.0156
Median [IQR]	10.0 [9.3–10.0]	3.5 [1.3–9.0]	
**Blood eosinophils (cells/µL)**			
Mean ± SD	404 ± 215	342 ± 197	0.138
Median [IQR]	420 [238–578]	290 [213–483]	
**ACT** *			
Mean ± SD	20.3 ± 5.9	23.9 ± 2.3	0.375
Median [IQR]	23 [18–24]	25 [24–25]	

* ACT evaluated only in patients with asthma (n = 7). Abbreviations: NPS: Nasal Polyp Score; SNOT-22: Sinonasal Outcome Test-22; VAS: visual analogue scale; IQR: interquartile range; SD: standard deviation.

**Table 3:** Multidimensional treatment response after 12 months of dupilumab therapy.

Patient	NPS Reduction ≥ 1	SNOT-22 Reduction ≥ 8.9	Olfactory VAS Improvement ≥ 3	ACT Increase ≥ 3 *	No Rescue Treatment	Number of Domains	Response Category
P01	Yes	Yes	No	No	Yes	3	Moderate
P02	Yes	Yes	No	NA	Yes	3	Moderate
P03	Yes	Yes	Yes	Yes	Yes	5	Excellent
P04	Yes	Yes	Yes	No	Yes	4	Excellent
P05	Yes	Yes	Yes	No	Yes	4	Excellent
P06	Yes	Yes	No	No	Yes	3	Moderate
P07	Yes	Yes	Yes	NA	Yes	4	Excellent
P08	Yes	Yes	No	Yes	Yes	4	Excellent
P09	Yes	Yes	Yes	NA	Yes	4	Excellent
P10	Yes	Yes	Yes	Yes	Yes	5	Excellent

* ACT evaluated only in patients with asthma. Abbreviations: NPS: Nasal Polyp Score. SNOT-22: Sinonasal Outcome Test-22. VAS: Visual Analogue Scale. ACT: Asthma Control Test. NA: not applicable.

## Data Availability

The data supporting the findings of this study are available from the corresponding author upon reasonable request. Data are not publicly available due to privacy and ethical restrictions.

## Abbreviations

The following abbreviations are used in this manuscript:

ACT	Asthma Control Test
CRSwNP	Chronic Rhinosinusitis with Nasal Polyps
ESS	Endoscopic Sinus Surgery
N-ERD	NSAID-Exacerbated Respiratory Disease
NPS	Nasal Polyp Score
SNOT-22	Sinonasal Outcome Test-22
VAS	Visual Analogue Scale
